# Developmental Differences in Cortical Activation During Action Observation, Action Execution and Interpersonal Synchrony: An fNIRS Study

**DOI:** 10.3389/fnhum.2020.00057

**Published:** 2020-03-03

**Authors:** Wan-Chun Su, McKenzie L. Culotta, Michael D. Hoffman, Susanna L. Trost, Kevin A. Pelphrey, Daisuke Tsuzuki, Anjana N. Bhat

**Affiliations:** ^1^Department of Physical Therapy, University of Delaware, Newark, DE, United States; ^2^Biomechanics & Movement Science Program, University of Delaware, Newark, DE, United States; ^3^Department of Neurology & The UVA Brain Institute, University of Virginia, Charlottesville, VA, United States; ^4^Department of Language Sciences, Tokyo Metropolitan University, Tokyo, Japan; ^5^Behavioral Neuroscience Program, Department of Psychological & Brain Sciences, University of Delaware, Newark, DE, United States

**Keywords:** development, fNIRS, imitation, interpersonal synchrony, lateralization

## Abstract

Interpersonal synchrony (IPS) is an important everyday behavior influencing social cognitive development; however, few studies have investigated the developmental differences and underlying neural mechanisms of IPS. functional near-infrared spectroscopy (fNIRS) is a novel neuroimaging tool that allows the study of cortical activation in the presence of natural movements. Using fNIRS, we compared cortical activation patterns between children and adults during action observation, execution, and IPS. Seventeen school-age children and 15 adults completed a reach to cleanup task while we obtained cortical activation data from bilateral inferior frontal gyrus (IFG), superior temporal sulcus (STS), and inferior parietal lobes (IPL). Children showed lower spatial and temporal accuracy during IPS compared to adults (i.e., spatial synchrony scores (Mean ± SE) in children: 2.67 ± 0.08 and adults: 2.85 ± 0.06; temporal synchrony scores (Mean ± SE) in children: 2.74 ± 0.06 and adults: 2.88 ± 0.05). For both groups, the STS regions were more activated during action observation, while the IFG and STS were more activated during action execution and IPS. The IPS condition involved more right-sided activation compared to action execution suggesting that IPS is a higher-order process involving more bilateral cortical activation. In addition, adults showed more left lateralization compared to the children during movement conditions (execution and IPS); which indicated greater inhibition of ipsilateral cortices in the adults compared to children. These findings provide a neuroimaging framework to study imitation and IPS impairments in special populations such as children with Autism Spectrum Disorder.

## Introduction

Interpersonal synchrony (IPS) or the time-constrained movement coordination between two individuals is an important daily activity (Repp and Su, 2013). Some examples of IPS in daily life include walking or running at a matching pace with a partner, two people lifting a large or heavy object together, and children playing “follow the leader” games. Musicians often synchronize their actions while playing instruments in order to achieve harmony (Phillips-Silver and Keller, 2012). IPS has been studied across a variety of tasks spanning from simple finger tapping and reaching for objects (Rabinowitch and Knafo-Noam, 2015; Schmitz et al., 2017) to whole-body swaying/rocking (Sofianidis et al., 2012; Marsh et al., 2013) as well as walking (Wiltermuth and Heath, 2009). Yet, few studies have examined the developmental differences in IPS between children and adults. Moreover, the underlying neural mechanisms of IPS have not been well studied. In this study, we compared IPS performance and associated cortical activation patterns using functional near-infrared spectroscopy (fNIRS) between typically developing (TD) young adults and school-age children.

In the first 2 years of life, infants transition from imitation of discrete actions that are one-step and familiar to those that are multi-step and unfamiliar in nature (Jones, 2007). By 2 years, toddlers perform various sustained rhythmic actions such as walking, running, drumming, et cetera. (Clark and Phillips, 1993; Brakke et al., 2007). Preschoolers as young as two and a half years of age were able to scale their drumming tempo to that of their social partner (Kirschner and Tomasello, 2009). In a different study, young elementary school child-child pairs showed the lowest levels of IPS during joint drumming followed by middle school child-child pairs and lastly the young adult-adult pairs (Kleinspehn-Ammerlahn et al., 2011). The lower IPS levels of young children were attributed to their difficulties in adjusting to the variable nature of their partner’s hand coordination patterns (Kleinspehn-Ammerlahn et al., 2011). Infants and children will learn a variety of important social and adaptive skills by engaging in imitation and IPS with their social partners (Carpenter et al., 1998; Meltzoff, 2007). While short bouts of synchronization contribute to greater social bonding and pro-social behavior (Macrae et al., 2008; Tunçgenç and Cohen, 2016a,b), long-term exchanges of parent-child synchrony experiences will help develop secure attachments with caregivers (Isabella and Belsky, 1991). Children who engaged in more synchronous clap-tap actions had more prosocial behaviors towards their peers than those who had less synchronous actions (Tunçgenç and Cohen, 2016b). A broader meta-analysis of effects of IPS reported a medium-size effect on prosocial behaviors, a small-to-medium size effect on social bonding such as a greater sense of affiliation/similarity as well as better social cognition, for example, better memory of the partner (Mogan et al., 2017). By comparing the IPS performance and associated cortical activation during a novel and continuous reach to cleanup task between children and adults, the present study will highlight the developmental differences in IPS and related neural mechanisms.

While there are few studies describing neural substrates underlying IPS behaviors, various cortical structures have been implicated in imitation behaviors, and both behaviors may share similar neural substrates for their control (Bhat et al., 2017). Various cortical regions play an important role during the process of imitation (Iacoboni, 2005; Cattaneo and Rizzolatti, 2009). These include the frontal regions of the Inferior Frontal Gyrus (IFG) and ventral Premotor Cortex, the parietal regions such as the Inferior Parietal Lobule (IPL) and intraparietal sulcus of the parietal lobe, specifically, the Inferior Parietal Gyrus, Supramarginal Gyrus, and Angular Gyrus, and the temporal regions, specifically, the Superior Temporal Sulcus (STS; Iacoboni, 2005; Cattaneo and Rizzolatti, 2009). The STS responds more to biological than nonbiological stimuli and is thought to encode biological motion rather than the superficial characteristics of moving stimuli (Pelphrey et al., 2003a). Greater bilateral STS activation was found during imitation than action observation and execution suggesting that it provides a visual description of observed actions and compares the observed movements to that of planned actions (Molenberghs et al., 2010). On the other hand, IFG and IPL regions are said to be more active during the observation and imitation of goal-directed, object-related actions (Iacoboni, 2005; Pokorny et al., 2015). IPL may contribute to the motor planning aspects of imitated actions (De Renzi et al., 1983; Fontana et al., 2012); while IFG is said to be responsible for processing the goals of the action (Koski et al., 2002). It is important to note that during imitation these regions do not work in isolation and instead interact with each other as well as other brain regions such as the dorsolateral prefrontal cortices, premotor cortices, primary and supplementary/pre-supplementary motor cortices, cingulate/insular cortices, cuneus/precuneus as well as subcortical structures such as the cerebellum and putamen to form an important imitation network (Gazzola and Keysers, 2009; Iacoboni, 2009). Given the important role of the aforementioned cortical regions for imitation performance, in the present study, we will assess their contributions to components of IPS behaviors including action observation, action execution, and IPS itself during a continuous reach to cleanup task in both adults and children.

Original studies comparing cortical activation during action observation, execution, and imitation have reported similar levels of activation across all three tasks (Cattaneo and Rizzolatti, 2009; Molenberghs et al., 2010). However, recent studies have reported a more variable level of activation and lateralization across observation, execution, and imitation tasks. In terms of level of activation, one study reported greatest cortical activation during action imitation followed by action execution and lowest activation during action observation (Aziz-Zadeh et al., 2006) while another study found that cortical activation was greater during action execution and imitation than action observation (Reynolds et al., 2019). Multiple studies have also reported greater cortical activation during action imitation than action execution and observation (Hamzei et al., 2016; Brihmat et al., 2017; Gatti et al., 2017). However, the aforementioned patterns of activation differ depending on the regions of interest (ROIs). For example, Montgomery et al. (2007) found that IPL and IFG were more active during action imitation and execution compared to action observation whereas STS activation was greater during action imitation compared to action observation and execution. Varying patterns of hemispheric lateralization have also been reported for imitation behaviors. One of the original studies by Aziz-Zadeh et al. (2006) suggested that imitation control is more bilateral in nature. Other studies had shown that during action imitation STS activated bilaterally whereas IFG and IPL activation was more variable depending on the nature of the task (Mühlau et al., 2005; Montgomery et al., 2007; Gatti et al., 2017). During imitation of gesture or goal-directed actions, Mühlau et al. (2005) and Montgomery et al. (2007) found greater activation over left than right IPL but similar IFG activation between hemispheres, while Gatti et al. (2017) found greater activation in the right precentral gyrus and right IFG compared to their left homologues. In spite of the variable findings of past fMRI studies, they do offer some evidence for how the different cortical regions play a role during imitation and this could perhaps extend to IPS behaviors as well. However, the fMRI environment limits the study tasks to simple hand gestures without face-to-face social interactions. We still do not know if the aforementioned findings can be generalized to complex, everyday motor tasks within naturalistic social contexts.

fNIRS is a fairly novel neuroimaging technique that measures cerebral hemodynamics similar to fMRI, the gold-standard of neuroimaging (Lloyd-Fox et al., 2010). But unlike fMRI, which requires the participant to lie still in a narrow scanning bore, fNIRS only restrains a participant through a cap on the head and allows for measurements in the presence of movement as well as face-to-face interactions. Given its advantage to tolerate motion artifacts, fNIRS has been used to study cortical activation across various movements of walking (Holtzer et al., 2019), playing a dance video game (Tachibana et al., 2011), as well as free arm movements during face to face interactions with others (Egetemeir et al., 2011). Moreover, with its greater temporal resolution compared to fMRI (Lloyd-Fox et al., 2010), fNIRS does a better job of detecting the onset and features of the hemodynamic response (Hong et al., 2018; Khan et al., 2018). This ability to distinguish features of the hemodynamic response may help in identifying differences related to development as well as neuropathology. Other sophisticated applications of fNIRS include the use of fNIRS-based hemodynamic responses to facilitate human-computer interaction (Naseer and Hong, 2015) as well as multimodal use of fNIRS and EEG (Ge et al., 2017, 2019). Specifically, greater STS activation, as well as the larger amplitude of EEG-based evoked response potentials, have been reported during observation of intentional grasping compared to meaningless grasping (Ge et al., 2019). A handful of studies have examined cortical activation during naturalistic face-to-face IPS and social cooperation/competition using fNIRS (Egetemeir et al., 2011; Bolling et al., 2013; Liu et al., 2015; Bhat et al., 2017). During a cooperation/competition game, the cooperators showed greater right IFG activation compared to the competitors (Liu et al., 2015). Similarly, adults showed greater IPL activation during joint action with a partner during a table-setting task compared to solo table-setting motions (Egetemeir et al., 2011). We have replicated the work of Egetemeir et al. (2011) in healthy adults by comparing lateral cortical activation during observation, execution, and synchronization of a reach and clean up task (Bhat et al., 2017). We too found greater activation in cortical regions of STS, IFG, and IPL during action execution and IPS compared to action observation. More importantly, right IFG and IPL regions were more active during IPS than the action execution condition. We concluded that the action execution condition led to more left-lateralized cortical activation whereas the IPS condition led to more bilateral cortical activation suggesting an important role for the right frontoparietal networks during IPS behaviors.

Considering the important role of IPS in facilitating social development, it would be valuable to study developmental differences in IPS and associated patterns of activation. To date, few studies have compared IPS behaviors during naturalistic reaching tasks as well as the underlying brain activation patterns between TD adults and TD children to describe the developmental differences in IPS. Therefore, in this study, we aimed to investigate the differences in brain activation between TD adults and TD children as they observed, executed, and synchronized actions during a reach-cleanup task. We hypothesized that the quality of IPS in TD children would differ from TD adults. Specifically, we expected the level of IPS to be lower and patterns of cortical activation to somewhat differ between adults and children. However, we expect both groups to have greater bilateral activation during the IPS condition compared to action execution. Thirdly, we also expected synchrony performance to correlate with cortical activation.

## Materials and Methods

### Participants

Seventeen TD school-age children (mean age ± SE: 10.82 ± 0.69, 11 males and six females) and 15 TD adults participated in this study (mean age ± SE: 22.6 ± 0.7, eight males and seven females, *p* < 0.001 for the age difference between groups, no gender-based differences between groups, *p* > 0.1, Table 1). Individuals were recruited through word of mouth, online postings in local listservs as well as fliers in the community. As a first step, we completed screening interviews with potential participants to exclude individuals with any known neurological or psychiatric diagnoses, or those taking psychotropic medications, or any other difficulties that would prevent them from performing the study tasks. All participants had normal or corrected to normal vision. Based on a standard handedness questionnaire (Coren, 1992), 15 of the child participants were found to be strongly right-handed, while two children were moderately left-handed. Fourteen adult participants were strongly right-handed with one adult being weakly right-handed (Table 1). The activation patterns of the two moderately left-handed children and the weakly right-handed adult were similar to the group results as all had consistently used their right hand for completing the task; hence, their data have been retained following data analysis.

**Table 1:** Demographic and developmental/cognitive data.

Characteristics	Child (*n* = 17)	Adult (*n* = 15)
	Mean ± SE	Mean ± SE
Age	10.82 ± 0.69*	22.60 ± 0.70
Gender	11 male, 6 female	8 male, 7 female
Ethnicity	13 C, 1 A, 1 AI, 2 AC	12 C, 2 A, 1 Af
Handedness	15 R, 2 L	14 R, 1 L
VABS-II (SS)	110.29 ± 2.92	111.07 ± 2.53
Communication (SS)	109.82 ± 2.88	105.47 ± 1.65
Daily living (SS)	110.41 ± 3.08	110.07 ± 2.31
Socialization (SS)	106.53 ± 3.18	106.53 ± 2.05

VABS-II, Vineland Adaptive Behavior Scale-2nd Edition; SS, standard score; SE, Standard error; M, Male, F, Female; C, Caucasian, A, Asian; AI, American Indian; AC, Asian-Caucasian; Af, African American; R, right; L, left. *Indicates a significant difference between groups.

All participants completed the Vineland Adaptive Behavioral Scales (Volkmar et al., 1987) to provide measures of socialization (averaged standard score ± SE: children = 106.53 ± 3.18; adult = 106.53 ± 2.05, group difference: *p* > 0.1), communication (children = 109.82 ± 2.88; adult = 105.47 ± 1.65, group difference: *p* > 0.1), daily living skills (children = 110.41 ± 3.08; adult = 110.07 ± 2.31, group difference: *p* > 0.1) as well as overall adaptive functioning (children = 110.29 ± 2.92; adult = 111.07 ± 2.53, group difference: *p* > 0.1). Both groups showed typical levels of subdomain and overall adaptive functioning with no significant differences between groups (Table 1). The University of Delaware Institutional Review Board (IRB) approved this study protocol. Procedures were carried out in accordance with the recommendations of our IRB (IRB protocol id #: 1227966-1). All adult participants gave written informed consent, the parents of child participants approved their child’s participation, and the children gave their written assent as well, in accordance with the Declaration of Helsinki (as of 2008), prior to participation.

### Experimental Procedures

**Figure 1:**
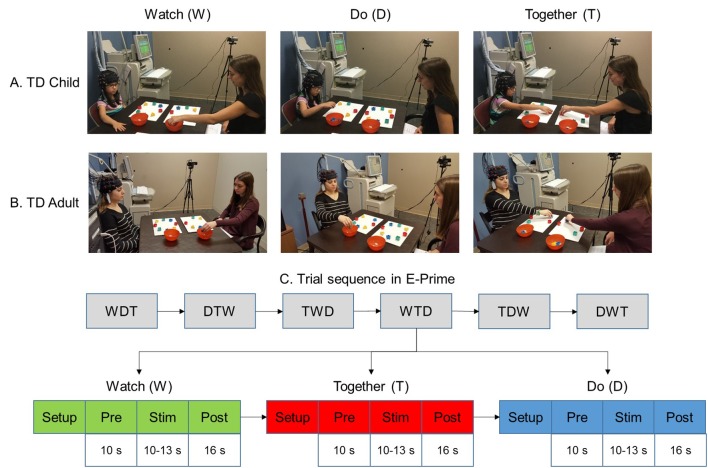
Experimental setup **(A,B)** and task sequence **(C)**. Written permission for publication of participant pictures has been taken.

Each participant and tester sat at a table facing each other to complete a reach to the cleanup task using a randomized blocked design (Bhat et al., 2017). Two 3 × 3 probes embedded in a cap were placed on the participant’s head (Figures 1A,B). Eight colored blocks were placed on a mat in a circular manner in front of both, the participant and the tester. Participants were asked to clean up the blocks off the mat into a bowl placed on the right using their right hand only. The participant completed three conditions: WATCH, DO, and TOGETHER (Figures 1A–C). During the WATCH condition, the participant observed the tester pick up the blocks in a sequential manner and put them into the container. Adults generally paid attention to the task; however, to ensure that the children paid attention during the WATCH trials, we asked them to focus on the pattern of cleanup. Before the trial, children were asked to pay attention to how the cleanup was performed. After a WATCH trial was completed, they were asked, “Which block did I pick up first?” Or “which block did I pick up last?” Or “how did I clean up the blocks?” For the DO condition, the participants cleaned up all the blocks in a sequence of their choice. In the TOGETHER condition, the tester led the block cleanup in random order while the participant followed by picking up the same block as the tester. No questions were asked after completing the DO and TOGETHER conditions. The participant was asked to use their right hand; while the tester used their left hand. The adults completed a total of 24 trials (eight trials per condition) whereas the children completed a total of 18 trials (six trials per condition). The stimulation period ranged between 10 and 13 s [Duration in seconds (Mean ± SE) in adults: *W* = 11.5 ± 0.18; *D* = 11.2 ± 0.3; *T* = 13.8 ± 0.6 and duration in children: *W* = 10.6 ± 0.2; *D* = 10.3 ± 0.4; *T* = 13.6 ± 0.6; *p* > 0.1 for group differences]. A 10-s pre-stimulation and a 16-s post-stimulation period were included to account for any baseline drifts in the fNIRS signal and to allow the hemodynamic response to return to baseline before starting the next trial. During baseline periods, the participants were asked to focus on a cross-hair on the front wall and remain as still as possible.

### Data Collection

**Figure 2:**
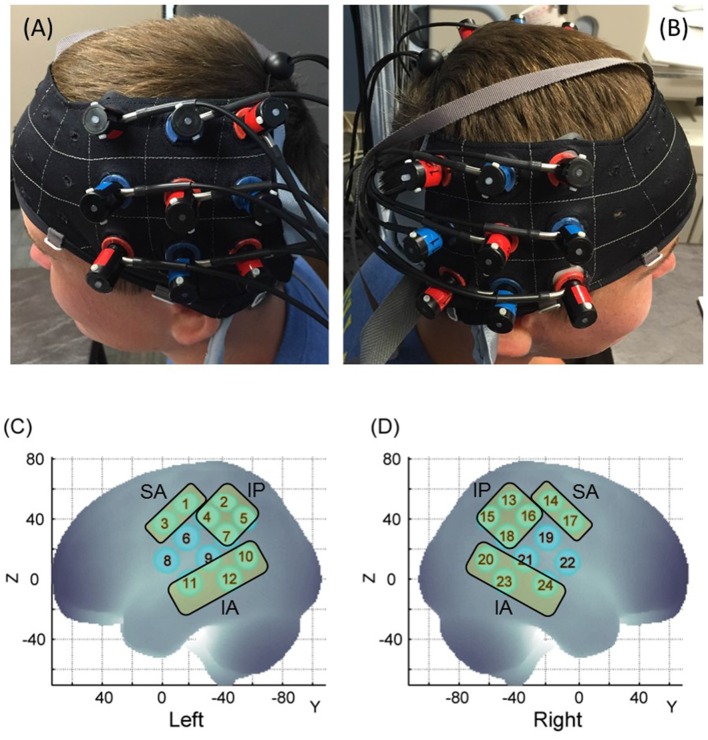
Probe placement **(A,B)** and spatial registration output **(C,D)**. Written permission for publication of participant pictures has been taken.

The hemodynamic changes over the ROIs were recorded using the Hitachi ETG-4000 system (Hitachi Medical Systems, Tokyo, Japan), with a sampling rate of 10 Hz. Two 3 × 3 probe sets, consisting of five infrared emitters and four receivers (i.e., 24 channels), were positioned over bilateral frontoparietal and temporal regions. Each adjacent pair of probes that were 3 cm apart were an emitter and receiver of two wavelengths of infrared light (695 and 830 nm). The middle column of the probe set was aligned with the tragus of the ear and the lowermost row of the optode set was aligned with the T3 position of the International 10-20 system (Klem et al., 1999; Figures 2A,B).

The infrared light passed through the skull creating a banana-shaped arc and reached the cortical area approximately below the midpoint of the two probes. The attenuation of infrared light was used to calculate the changes in concentrations of oxygenated (HbO_2_) and deoxygenated hemoglobin (HHb) chromophores per channel using the Modified Beer-Lambert Law. Based on results from previous studies, an increase in HbO_2_ concentration and a decrease in HHb concentration were expected with increased brain activation within a certain ROI (Lloyd-Fox et al., 2010).

E-Prime presentation software (version 2.0) was used to trigger the Hitachi fNIRS system. The entire session was videotaped using a camcorder that was synchronized with the Hitachi fNIRS system.

### Spatial Registration Approach

During the 3D registration process, each child was asked to remain in a still and upright position. The 3D locations of the standard cranial landmarks (nasion, inion, left and right preauricular points, and the Cz position of the International 10-20 system) as well as 3D locations of each probe in the probe set were recorded w.r.t. a reference coordinate system using the ETG-4000 3D positioning unit. These 3D coordinates saved in a text file format for each participant were run through MATLAB codes developed by the sixth author. The anchor-based, spatial registration method developed by Tsuzuki et al. (2012) was used to transform the 3D spatial location of each channel from the reference coordinate system to the Montreal Neurological Institute (MNI)’s coordinate system (see Figure 2, Supplementary Table S1). Structural information from an anatomical database (Okamoto et al., 2004) was used to provide estimates of channel positions within a standardized 3D brain atlas (Tsuzuki et al., 2012). The estimated channel locations were anatomically labeled using the LONI Probabilistic Brain Atlas (LPBA; Shattuck et al., 2008). Note that each run includes position data from all participants within one group to obtain the average MNI coordinates for each channel. Based on the regions covered by our channels, we assigned the 24 channels to three ROIs for the children. Similarly, please refer to Bhat et al. (2017) for the channel assignments in adults across the same three ROIs.

The three ROIs included: (i) the Superior Anterior region (SA) which included channels over the inferior/middle frontal gyrus or IFG and pre-central gyrus (or frontal cortices, left: channels 1 and 3, and right: channels 14 and 17 channels, see Figures 2C,D); (ii) the Inferior Posterior region (IP) which included channels over the post-central gyrus, supramarginal gyrus, and angular gyrus (or the inferior posterior parietal cortices or IPL, left: 2, 4, 5, 7 channels and right: 13, 15, 16, 18 channels, see Figures 2C,D); and (iii) the Inferior Anterior region (IA) which included channels over the middle and superior temporal gyrus (or superior temporal cortices or STS, left: 10, 11, 12 channels and right: 20, 23, 24 channels, see Figures 2C,D). These three ROIs separated the three cortical regions we described earlier. Channels 6, 8, 9 (left) and 19, 21, and 22 (right) were excluded due to spatial uncertainty. To be clear, spatial uncertainty occurred when either one of the two homologous channels did not fall within the same ROI for a particular group. Another reason for spatial uncertainty was when any given channel did not cover 60% or more of the assigned ROI and instead covered multiple ROIs evenly, for example, 50% IPL and 50% IFG; such channels were excluded. In this way, we were able to consistently assign 18 out of the 24 channels to one of the aforementioned ROIs in both groups. Note: Supplementary Table S1 in the “Supplementary Materials” section shows the channel assignment in children and refers to Bhat et al. (2017) for channel assignment in adults.

### Data Processing

**Figure 3:**
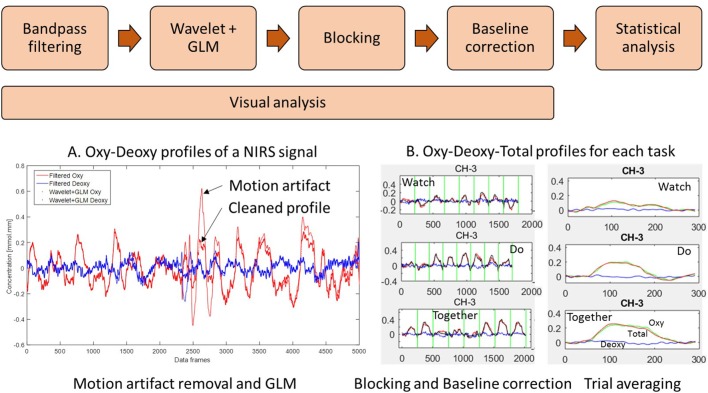
Data processing workflow: **(A)** filter, wavelet and general linear model (GLM) of NIRS signal and **(B)** trial-by-trial view and Average view of Oxy Hb (HbO_2_), Deoxy Hb (HHb), and Total Hb (HbT) profiles for a given channel. (W, D, T) from 5 s before to 24 s after the start of stimulation. Data have been averaged across trials and participants.

We have written our own customized MATLAB programs that incorporate functions from open-source software such as Hitachi POTATo (Sutoko et al., 2016) and Homer-2 (Huppert et al., 2009) to analyze the data from the ETG system (see data processing steps in Figure 3). The sampling frequency of the fNIRS system was 10 Hz (i.e., 10 data frames per second were collected). Data from each channel was first band-pass filtered between 0.01 and 0.5 Hz using the Fast Fourier Transform (FFT) method to remove lower or higher frequencies associated with body movements and other dynamic signals/tissue such as respiration, heart rate, skin blood flow, etc. The low-pass filter removes physiological noises related to respiration and fast cardiac oscillations and high-frequency instrument noise, whereas the high-pass filter minimizes the low-frequency drift from the data. To remove motion artifacts, we used the wavelet method (Sato et al., 2006; Huppert et al., 2009) which is one of the most robust methods for motion artifact removal (Hu et al., 2015). In this method, it is assumed that the measured signal is a linear combination of the desired signal and the undesired artifacts. The number of levels for wavelet decomposition is calculated by taking the logarithm of the number of data points using a base of 2. For our dataset, this value was 14. By applying the 1-D discrete wavelet transform to the signal from each channel, details of the signal are estimated as approximation coefficients. Assuming that the detail wavelet coefficients have a Gaussian distribution, outliers in the distribution correspond to the coefficients related to the motion artifacts. To identify the motion artifacts/outliers, an “iqr” parameter of 1.5 was used. The coefficients greater than the iqr parameter times the interquartile range of the data are typically associated with motion artifacts, and hence, they were set to zero to remove such artifacts. The inverse discrete wavelet transform is applied and the signal is reconstructed. Next, the General Linear Model (GLM) was implemented using a HOMER-2 MATLAB function. GLM estimated the hemodynamic response function using Gaussian basis functions and a 3^rd^ order polynomial drift regression (Huppert et al., 2009). To correct the baseline drifts, the linear trend between the pre-trial baseline and the post-trial baseline was calculated and subtracted from values in the stimulation period as implemented in Hitachi POTATo (Sutoko et al., 2016). Average HbO_2_ and HHb values were obtained for the stimulation period of each trial. The range of HbO_2_ data was significantly greater than the HHb data. Moreover, HbO_2_ profiles have a greater signal to noise ratio compared to HHb and therefore consistent with fNIRS literature, we have reported HbO_2_ profiles (Sato et al., 2006). The data were plotted and saved at each step. We visually screened the plotted figures at each step of the analysis to exclude channels/trials. We excluded channels with poor contact (flat lines) or persistent motion artifacts or obvious outliers compared to the other similar trials from each condition. Nearly 6.7% of data from children and 19.2% of data from adults have been removed using these criteria. In the “Supplementary Materials” section, we have also provided a visual representation of the second-to-second HbO_2_ profile for each group (Supplementary Figure S1: Adults, Supplementary Figure S2: Children), each condition, and each channel for the entire period (pre-baseline, stimulation, and post-baseline). The pink vertical line denotes the start of the stimulation period and the data shown to the right of the pink line are the 240 frames across stimulation (10–13 s) and post-stimulation baseline (14–11 s) periods.

#### Video Data Coding

Two trained student researchers scored the session videos in order to exclude trials with significant errors. Inter-rater reliability of above 85% and intra-rater reliability of above 90% were achieved for all scores between the primary and secondary coder for 20% of the video dataset. The trials were excluded from data analysis if the participants did not follow task instructions, had significant body movements unrelated to the task, or spoke to the tester during the trials. A three-point scale was used to code the synchrony and motor quality during IPS. Spatial synchrony scores were rated from 1 to 3 with 1 = Picked up incorrect blocks for more than once, 2 = Picked up the incorrect block once, and 3 = Picked up all blocks correctly. Temporal synchrony scores were rated from 1 to 3 with 1 = More than one block delay, 2 = One-block delay and 3 = Perfect synchrony. Motor errors were defined as two-hand use, picking up more than one block at the same time, slippage when picking or placing, while the motor scores were rated with 1 = More than 4 errors, 2 = 2–4 errors, 3 = 0–1 error. The number of additional movements during the stimulation period was also coded. Ultimately, we eliminated 7.0% of the overall child data and 1.1% of the overall adult data due to persistent motion artifacts. Specifically, in the TD child group, 7.3% of Watch, 9.8% of Do, and 3.9% of Together or 1–2 trials out of the six trials for each condition were excluded. In the TD adult group, 0% of Watch, 0% of Do, and 3.3% of Together or approximately 1–2 out of the eight trials per condition were excluded.

### Statistical Analyses

To avoid multiple channel-wise comparisons, we averaged data across channels within the same ROI based on our spatial registration output (Figures 2C,D show the six ROIs and constituent channels). All participants primarily moved their right hand during the task, therefore, right hemisphere activation should be considered ipsilateral, and left hemisphere activation would be contralateral to the moving arm of our participants. We determined levels of activation for six ROIs including the left and right superoanterior (SA), inferoposterior (IP), and inferoanterior (IA) regions (Figure 2 shows the different ROIs). Using IBM SPSS (SPSS Inc., Chicago, IL, USA), we conducted repeated-measures ANOVA with within-group factors of group (children, adult), condition (Watch, Do, Together), hemisphere (left, right), and ROI (SA, IP, IA) and a between-group factor of group (child, adult) for average HbO_2_ values. Greenhouse-Geisser corrections were applied when our data violated the sphericity assumption based on Mauchly’s test of sphericity. For multiple *post hoc* comparisons, we have used the False Discovery Rate (FDR) method proposed by Singh and Dan (2006) for multichannel fNIRS data. We specifically used the Benjamin-Hochberg method wherein unadjusted *p*-values are rank-ordered from low to high. Statistical significance is declared if the unadjusted *p*-value is less than the *p*-value threshold. *p*-threshold was determined by multiplying 0.05 with the ratio of unadjusted *p*-value rank to the total number of comparisons (*p*-threshold for i^th^ comparison = 0.05 × i/n; where n = total number of comparisons). Paired *t*-tests were used to examine group differences in behavioral data including temporal/spatial synchrony score, motor score, and additional movements.

## Results

### Quality of IPS Behaviors

Paired *t*-tests showed that children had lower spatial synchrony scores (Mean ± SE = 2.67 ± 0.08) compared to adults (2.85 ± 0.06, *p* = 0.03). Similarly, children had lower temporal synchrony scores (2.74 ± 0.06) compared to adults (2.88 ± 0.05, *p* = 0.04) indicating more errors in the children vs. the adults. There were no significant group differences in terms of motor pattern errors (Children: 2.97 ± 0.01; Adults: 2.97 ± 0.01, *p* > 0.1) or additional movements (Children: 1.41 ± 0.43; Adults: 0.73 ± 0.23, *p* > 0.1; Table 2).

**Table 2:** The quality of interpersonal synchrony (IPS) in the TD children and adults.

Video coding variables	Child (Mean ± SE)	Adult (Mean ± SE)
Spatial IPS	2.67 ± 0.08*	2.85 ± 0.06
Temporal IPS	2.74 ± 0.06*	2.88 ± 0.05
Motor score	2.97 ± 0.01	2.97 ± 0.01
Do condition	2.96 ± 0.02	2.97 ± 0.01
Together condition	2.99 ± 0.01	2.97 ± 0.01
Additional movements	1.41 ± 0.43	0.73 ± 0.23

*Indicates a significant difference between groups.

### Cortical Activation During IPS

The group × condition × hemisphere × region four-way repeated ANOVA revealed a significant main effect of group (*F*_(1,119)_ = 7.6, *p* = 0.007), condition (*F*_(2,229.4)_ = 145.2, *p* < 0.001), hemisphere (*F*_(1,119)_ = 30.7, *p* < 0.001), and region (*F*_(1.8,220.2)_ = 132.3, *p* < 0.001) as well as two-way interactions between group × condition (*F*_(1.8,217.2)_ = 17.6, *p* < 0.001), group × hemisphere (*F*_(1,119)_ = 12.4, *p* < 0.001), condition × hemisphere (*F*_(1.7,203.5)_ = 43.4, *p* < 0.001), condition × region (*F*_(3.6,422.1)_ = 18.2, *p* < 0.001), as well as three-way interactions between group × condition × hemisphere (*F*_(1.4,170.5)_ = 5.5, *p* = 0.01) and condition × hemisphere × region (*F*_(3.3,396.2)_ = 4.9, *p* = 0.04). *Post hoc* analyses were focused on two three-way interactions of condition × hemisphere × region and group × condition × hemisphere (Table 3 shows the Mean and SE of HbO_2_ concentration values, Table 4 shows significant *p*-values and direction of effects, and Figure 7 shows channel specific activation data).

**Table 3:** The mean and standard error (SE) of activation based on HbO_2_ concentration values.

Group activation data	Watch		Do		Together	
	Mean	SE	Mean	SE	Mean	SE
**TD child**
Left hemisphere
SA/fronto-parietal	0.007	0.004	0.052	0.004	0.053	0.005
IA/temporal	0.020	0.006	0.055	0.006	0.052	0.007
IP/inferior parietal	−0.006	0.004	0.006	0.005	0.007	0.005
Right hemisphere
SA/fronto-parietal	0.011	0.005	0.041	0.005	0.053	0.006
IA/temporal	0.032	0.007	0.030	0.006	0.040	0.007
IP/inferior parietal	−0.008	0.004	−0.003	0.004	0.002	0.005
**TD adult**
Left hemisphere
SA/fronto-parietal	0.001	0.004	0.079	0.010	0.076	0.009
IA/temporal	0.012	0.004	0.087	0.008	0.091	0.008
IP/inferior parietal	−0.009	0.003	0.051	0.006	0.045	0.006
Right hemisphere
SA/fronto-parietal	0.003	0.004	0.038	0.007	0.061	0.007
IA/temporal	0.007	0.004	0.033	0.005	0.042	0.005
IP/inferior parietal	−0.013	0.003	−0.001	0.003	0.014	0.004

**Table 4:** A listing of significant *p*-values and direction of the effect during *post hoc* *t*-tests.

Comparison	Significant *p*-values	Direction of effect
**Group differences**
Watch, R hemisphere	<0.001	Child > Adult
Do, L hemisphere	<0.001	Adult > Child
Together, L hemisphere	<0.001	Adult > Child
**Task-related differences**
Group × condition × hemisphere
(regions were pooled)
Adult, L hemisphere	<0.001	D & T > W
Adult, R hemisphere	<0.001	T > D > W
Child, L hemisphere	<0.001	D & T > W
Child, R hemisphere	<0.010	T > D > W
Condition × hemisphere × region
(groups were pooled)
Left SA, IA & IP	<0.001	D & T > W
Right SA, IA & IP	<0.05	T > D > W
**Hemispheric differences**
Group × condition × hemisphere
(regions were pooled)
Adult, Do	<0.001	L > R
Adult, Together	<0.001	L > R
Child, Do	<0.001	L > R
Condition × hemisphere × region
(groups were pooled)
SA, IA, & IP ROIs for Do	<0.001	L > R
IA & IP ROIs for Together	<0.001	L > R
**Regional differences**
Watch, L & R hemispheres	<0.010	IA > SA > IP
Do, L & R hemispheres	<0.001	SA & IA > IP
Together, L hemisphere	<0.001	SA & IA > IP
Together, R hemisphere	<0.010	SA & IA > IP

**Figure 4:**
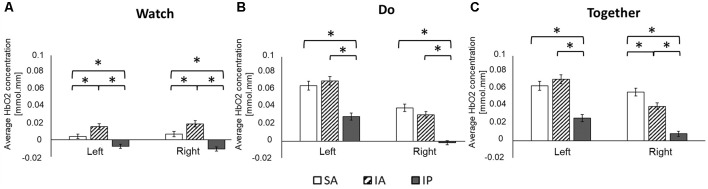
Regional differences (**A**: Watch; **B**: Do; **C**: Together) in average HbO_2_ concentration. *Indicates a significant difference between regions.

**Figure 5:**
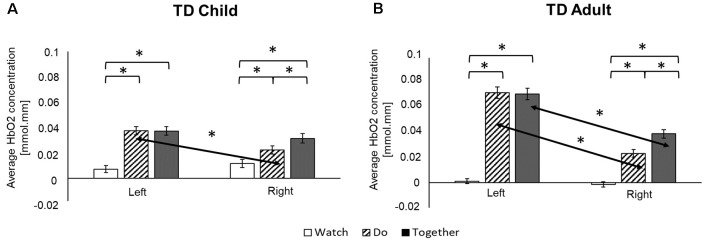
Task-related and hemispheric differences for typically developing (TD) child **(A)** and adult **(B)** in average HbO_2_ concentration. *Indicates a significant difference. Arrows highlight hemispheric differences.

**Figure 6:**
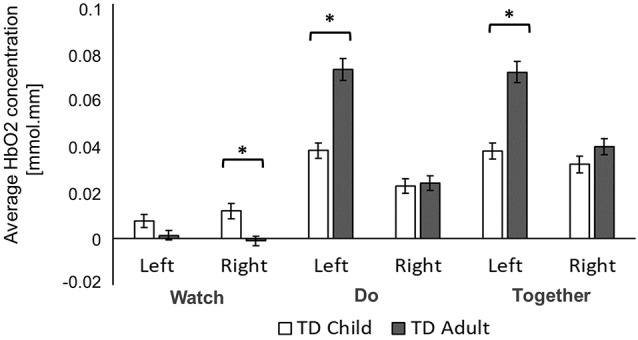
Group differences in average HbO_2_ concentration. *Indicates a significant difference between groups.

**Figure 7:**
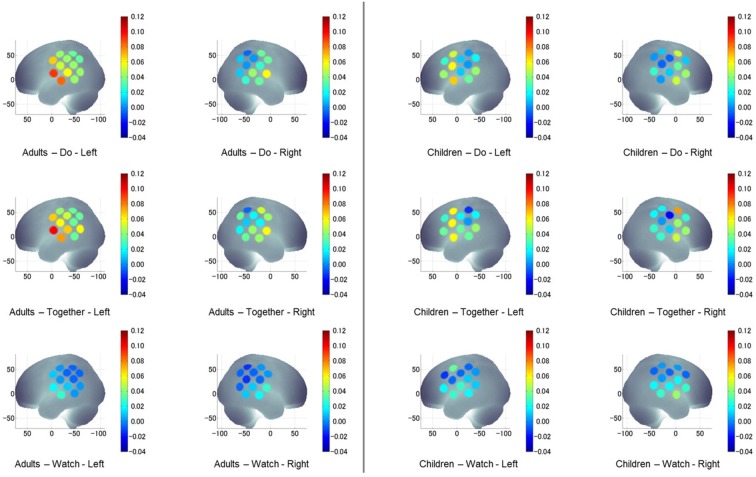
A visual representation of task-related, hemispheric, and group differences in channel activation (HbO_2_) during the stimulation period compared to its own baseline in both groups and all conditions.

#### Regional Differences in Both Groups

During the Watch condition, both groups had greater activation in the IA (STS) region (left and right ROIs) compared to SA (IFG) and IP (IPL) regions (*p*s ≤ 0.001). During the Do and Together conditions, both groups had greater SA (IFG) and IA (STS) activation compared to the IP (IPL) region for both left and right ROIs. Lastly, during the Together condition, both groups had greater activation in the right SA (IFG) than right IA (STS; *p*s < 0.01, Figures 4, 7).

#### Task-Related Differences in Both Groups

Both children and adults showed greater activation over the left and right ROIs during the Do and Together conditions compared to the Watch condition (*p*s < 0.05, Figures 5, 7). The differences between Do and Together conditions were only limited to the right hemisphere. Both groups showed greater activation in the right ROIs only (not left) during Together compared to the Do condition (*p*s < 0.05, Figures 5, 7).

#### Hemispheric Differences in Both Groups

Adults had greater activation in the left hemisphere compared to the right hemisphere during both movement conditions of Do and Together (*p*s < 0.001, see Figure 5’s left vs. right comparisons and Figure 7). However, children had greater activation in the left hemisphere compared to the right hemisphere for the Do condition only (*p* < 0.001, see Figure 5’s left vs. right comparison and Figure 7) but not the Together condition (*p* > 0.1).

#### Group Differences Across Tasks

During the Watch condition, children had greater activation in the right hemisphere than the adults (*p*s < 0.001, Figures 6, 7). During the Do and Together conditions, adults had greater left hemispheric activation than children (*p*s < 0.001, Figures 6, 7).

#### Correlation Between IPS Behaviors and Cortical Activation

For correlations between IPS behaviors and cortical activation, adults showed more significant correlations compared to children (number of correlations in children = 3 and adults = 12, Table 5). More specifically, the adult IPS spatial scores correlated with cortical activation in all three ROIs across all conditions. In addition, the adult IPS temporal scores correlated with SA and IP activation only during the Do and Together conditions. In slight contrast, the children’s IPS spatial scores correlated with right SA and left IP activation in the Do and Together conditions and their IPS temporal scores did not correlate with cortical activation.

**Table 5:** Correlation between IPS behaviors and cortical activation.

		IPS spatial			IPS temporal		
		Watch	Do	Together	Watch	Do	Together
**TD children**
SA	Left	ns	ns	ns	ns	ns	ns
	Right	ns	−0.36**	−0.40**	ns	ns	ns
IA	Left	ns	ns	ns	ns	ns	ns
	Right	ns	ns	ns	ns	ns	ns
IP	Left	0.23*	0.20*	0.38**	ns	ns	ns
	Right	ns	ns	ns	ns	ns	ns
**TD adult**
SA	Left	ns	0.30**	0.33**	ns	0.42**	0.44**
	Right	0.24**	ns	ns	ns	−0.21*	−0.28**
IA	Left	ns	−0.21*	ns	ns	ns	ns
	Right	ns	0.27**	0.33**	ns	ns	ns
IP	Left	ns	0.27**	0.34**	ns	0.28**	0.33**
	Right	0.23*	ns	0.20*	ns	ns	ns

The table presents *r* values using Spearman-rank correlations. **p < 0.01, *p < 0.05, ns: non-significant.

## Discussion

Previous fMRI studies of IPS have been limited to simple hand movements and unnatural environments. Using fNIRS, two studies have reported differences in cortical activation during IPS vs. solo action execution (Egetemeir et al., 2011; Bhat et al., 2017). However, no study has compared developmental differences in IPS performance as well as underlying cortical activation patterns between children and adults. The present study compared the cortical activation patterns between children and adults performing action observation, execution, and IPS during a naturalistic reach to the cleanup task. Consistent with our hypothesis, TD children had lower IPS than adults with children showing lower spatial and temporal synchrony scores compared to adults. However, the two groups did not differ in terms of motor pattern scores or other body movements. These findings suggest that while the accuracy of simple reaching motions was similar between adults and children, the ability to synchronize reaching motions with another individual was still developing between childhood and adulthood.

We found some similarities as well as differences between the cortical activation patterns of children and adults. First, both groups had greater cortical activation during the Do (execution) and Together (IPS) conditions compared to the Watch (observation) condition. More importantly, in the Together (IPS) condition, both groups had greater right hemispheric activation compared to the Do condition. In terms of regional similarities, in the Watch condition, both groups had greater activation in the IA (superior temporal or STS) region compared to the SA (inferior frontal or IFG) and IP (inferior parietal or IPL) regions for both hemispheres. During the Do and Together conditions, both groups had greater SA (IFG) and IA (STS) activation compared to the IP (IPL) region in both hemispheres. Lastly, during the Together condition, both groups had greater activation in the right SA (IFG) than the right IA (STS) region. However, we noted some differences in cortical activation patterns between children and adults. In terms of the within-group, hemispheric differences, adults had greater left hemispheric activation (than right) for both Do and Together conditions. However, in the TD children, this pattern was seen only for the Do condition with more bilateral activation in the Together condition. In terms of the between-group differences, during the Watch condition, TD children had greater right hemispheric activation compared to adults. Additionally, in the Do and Together conditions, the adults had greater left hemispheric activation than the TD children. Lastly, adult spatial synchrony scores correlated with cortical activation in all three ROIs and their temporal synchrony scores correlated with SA (IFG) and IP (IPL) activation only. In contrast, children’s spatial scores correlated with the right IFG and left IPL activation but not their temporal scores.

### IPS Improves Between Childhood and Adulthood

Children had lower spatial and temporal synchrony scores compared to adults suggesting lower IPS in children than adults. To our knowledge, only one study has compared developmental differences in IPS performance. During a joint drumming task, adult-adult dyads showed the highest levels of IPS and least within-individual, inter-limb variability followed by older child-child dyads and lastly the younger child-child dyads, who showed the lowest levels of IPS and greatest within-individual, inter-limb variability (Kleinspehn-Ammerlahn et al., 2011). It was posited that the greater variability in arm movements of younger children contributed to their action inconsistency and ability to synchronize with each other. Although we could not find other comparisons of IPS performance between children and adults, one study comparing visual-motor synchronization of children and adults to various rhythmic visual stimuli found that 7–8-year-old children showed more variability and longer periods of asynchrony compared to adults (Kurgansky and Shupikova, 2011). Similarly, in our study, children made more errors in mirroring their choice of block or were more off in their timing of reaching or cleanup motions compared to the adults as they synchronized their actions to an adult tester. While our behavioral coding did not reveal any group differences in motor errors, we do not know if there was greater reaching variability in our child participants because we did not capture the reaching trajectories of both groups. Although behavioral coding did not reveal any obvious differences in the attentional patterns of both groups, possibly the differences in the visuomotor mapping of one’s hand motions to that of the social partner could have contributed to the IPS differences between children and adults (Tahej et al., 2012).

### STS Region Is Important for Observing Human Actions

During the Watch condition, both groups had greater STS activation than any other ROIs; however, children had greater right STS activation than the adults. The adult portion of this study was conducted before the child portion of the study. From coding of adult data, we noticed that mere instruction to watch during the Watch condition did not lead to careful observation of the tester’s reaching motions. Hence, for the child group, in order to promote sustained attention, we asked the children to observe our motions carefully so that they could answer questions about how the task was completed at the end of the trial. Specifically, we asked questions, for example, “Which block was cleaned up first or last, etc.?” This may have contributed to the greater social attention as well as greater STS activation observed in the children vs. the adults. Nevertheless, the results were similar between the two groups in that both groups had predominant STS activation during the Watch condition compared to activation in other ROIs (Bhat et al., 2017). Multiple fMRI studies have reported significant STS activation during action observation tasks (Montgomery et al., 2007; Molenberghs et al., 2010; Gatti et al., 2017). The STS region is important for processing and distinguishing social information such as biological motion, goal-directed actions of others, and mutual social gaze (Pelphrey et al., 2003a,b; Pelphrey and Morris, 2006). Pelphrey et al. (2003a) showed greater STS activation during observation of human or robotic motions compared to non-biological, object-related motions. Our common finding of greater fNIRS-based activation in superior temporal cortices during action observation in both groups (adults and children) is consistent with findings from past fMRI studies. Additionally, during a computerized ball toss game involving healthy adults, fNIRS-based activation was increased within the STS region when observing biological motion within a social inclusion context vs. a social exclusion context (Bolling et al., 2013). Therefore, in agreement with multiple fMRI and the few fNIRS studies (Bolling et al., 2013; Bhat et al., 2017) we also found greater STS activation during social observation of other’s actions in both children and adults.

### Role of IFG, STS, and IPL During Goal-Directed Actions and Their Importance for Visuomotor Correspondence During Imitation and IPS

During the movement conditions of the reach to cleanup task i.e., Do and Together conditions, both groups had greater activation in the IFG and STS compared to IPL regions. Moreover, adult synchrony scores correlated with cortical activation in all three ROIs whereas children’s synchrony scores correlated with right IFG and left IPL activation only. Activation in the IFG was not very surprising because these regions are important for goal-directed movements (i.e., both Do and Together conditions required accurate reaching to objects; Cincotta and Ziemann, 2008). Similarly, during the self-selected motor task (i.e., the Do condition) we found temporal cortex activation (i.e., STS and middle temporal gyrus) in spite of no overt social interactions between the participant and tester. Testers were asked to avoid eye contact and overt social interactions with the participant during action execution. Additionally, we viewed the video data to remove any Do trials that involved social interactions; however, the mere presence of the tester may have contributed to some of the STS activations. Our findings somewhat fit with the current fMRI literature reporting significant STS activation during action imitation tasks compared to action execution and observation (Montgomery et al., 2007; Molenberghs et al., 2010). During object-based gesture tasks, bilateral STS activation was greater during action imitation compared to action execution and observation, which had similar activation levels (Montgomery et al., 2007). STS regions are said to provide a visual description of actions (Iacoboni, 2005). Molenberghs et al. (2010) suggested that STS is not merely registering the biological motions during imitation but also encoding the visuomotor correspondence between one’s own actions and that of the partner. An fMRI study measuring cortical activation during observation of congruent vs. incongruent actions between two individuals revealed greater STS activation in the incongruent than congruent condition further corroborating the idea that STS may indeed be encoding visuomotor correspondence between individuals when moving together (Shibata et al., 2011).

The STS region could be interacting with IFG and other regions to receive efference copies of the motor plans to match the performed actions with the visual descriptions of imagined or observed actions (Iacoboni, 2005; Molenberghs et al., 2010). In our study, cortical activation during IPS was more similar to that of activation during action execution (not action observation). Additionally, synchrony errors in both groups correlated most with the Do and Together conditions (nine correlations per condition) and lastly the Watch condition (three Watch correlations). We believe our findings show that the challenges of imitation/IPS control stem from the complexity of motor components and not so much the observation component. It is often reported in the literature that simpler imitative tasks require less cortical activation compared to complex motor tasks and imitation performance is inextricably linked to its motor requirements such as body parts/joints involved as well as action complexity (Gatti et al., 2017).

The IPL region is also said to play an important role in planning the kinematics and goals of solo and complementary gestures/actions (Buxbaum et al., 2006; Sacheli et al., 2015). Specifically, the left parietal lobe contributes to visuospatial planning of actions with its lesions resulting in more errors during meaningless actions due to their more complex planning requirements (Tessari et al., 2007). Transcranial magnetic stimulation of dorsal parietal cortices interfered with online adjustments of reach-grasp actions suggesting its role in integrating end goals and motor planning (Tunik et al., 2008). Similarly, left parietal cortex activation was also reported by Sacheli et al. (2015) when performing joint grasping tasks in order to encode shared goals of complementary actions. Taken together, our findings fit with past literature confirming the role of STS, IFG, and IPL regions for visuomotor correspondence during both solo and synchronous reach-grasp actions.

### Greater Left-Hemispheric Activation During Movement Tasks With Adults Showing More Left Lateralization

In general, during unilateral movement tasks of reach and cleanup, the two groups differed in that the adults had greater left lateralization than the children. To be clear, even when children used the right arm to complete the task there was perhaps some low-level “mirror” activation present in the homologous muscles of the left arm. This inability to suppress activity in the ipsilateral sensorimotor cortices has been reported in past studies comparing unilateral motor tasks between adults and children (Mayston et al., 1999; Huo et al., 2011). Mirror movements have been reported in children below 10 years of age but will diminish into adolescence and adulthood (Connolly and Stratton, 1968; Nass, 1985). Studies using transmagnetic stimulation (TMS) and magnetoencephalography (MEG) showed that during unilateral finger movements young children had activation in both contralateral and ipsilateral motor cortices due to lack of transcallosal inhibition resulting in muscle activity in the homologous muscles of the less active arm. However, this pattern of bilateral activation was not seen in adolescents and adults (Mayston et al., 1999; Huo et al., 2011). It is not surprising to see similar cortical activation patterns in our study since 65% of our child sample is below 11 years of age.

### Greater Right Hemispheric Activation During IPS in Adults and Children

Both groups had increased right-hemispheric activation during the Together condition, compared to the Do condition, in spite of the right-handed nature of the reach-cleanup task. These results suggest that while there is left-lateralization during the Do condition (unilateral movements), IPS constraints led to more bilateral activation. These findings concur with a comprehensive meta-analytic review that showed activation of bilateral networks including frontal, premotor, parietal, and the temporo-occipital cortex during imitation (Caspers et al., 2010). Aziz-Zadeh et al. (2006) had participants observe, imitate, or execute unilateral finger movements with right or left hands to cues shown in the right or left visual field (hand moving or fixation cross). The imitation condition involved greater right inferior frontal and inferior parietal cortex activation in contrast to action execution, which mainly activated the contralateral primary visual and motor cortices. It was suggested that even during unilateral action imitation there is greater ipsilateral cortical activation compared to action execution, which is primarily contralateral in its control (Aziz-Zadeh et al., 2006). Similarly, Biermann-Ruben et al. (2008) found that imitation of biological hand movements led to greater right fronto-temporal activation compared to non-biological hand motions. Another group of fMRI studies has compared specular (mirrored—left-hand tester/video, right hand of subject) and anatomical imitation (both tester and subject use the identical hand for imitation, both use their right or left hands) and report greater bilateral or right hemispheric activation during specular imitation compared to anatomical imitation (Koski et al., 2003; Mengotti et al., 2015). In terms of fNIRS literature, few studies have reported greater bilateral activation during synchronous/cooperative actions with another partner (Egetemeir et al., 2011; Liu et al., 2015; Bhat et al., 2017). During a table-setting task, Egetemeir et al. (2011) reported greater activation in bilateral IPL regions during a joint action condition compared to the solo action or observation condition. Similarly, when two adults engaged in a cooperation game, the fNIRS patterns suggested that the partner who was actively following had greater right IFG activation compared to the partner who was a passive follower (Liu et al., 2015). In short, multiple studies have suggested that action imitation requires significant right/bilateral hemispheric activation beyond what is required during action execution. Consistent with the current literature, both children and adults in our study showed greater right/bilateral hemispheric activation during the IPS condition.

### Mechanistic Framework for IPS

In this section, we highlight the common components across the different frameworks explaining the underlying processes associated with IPS behaviors (Semin and Cacioppo, 2009; Iacoboni, 2009; Pineda, 2009; Vesper et al., 2010, 2017). When engaging in IPS, each partner must understand the shared task goal as well as each of their individual roles in the task. While the overall goals are shared and similar; each partner’s goals can be individual and distinct (Vesper et al., 2010). For example, when cleaning up blocks “together,” the common goal was to move matching blocks in-synchrony; however, each participant still had to identify the appropriate block, pick, and place it in the container. In fact, it has been shown that partners will forego the quality of their own actions to complement and support the broader goal of moving with their partner (Schmitz et al., 2017). Some examples of how partners modify their actions for accomplishing the shared goal include changes in action speed or salience or workspace (Vesper et al., 2009, 2010, 2011; Schmitz et al., 2017). In terms of cortical regions, the IFG region is considered important for goal understanding during goal-directed actions such as reaching (Fontana et al., 2012). Additionally, the interactions between IFG and IPL regions are important for motor planning and sensorimotor representations for anticipatory control of actions (Koski et al., 2002). Second, during IPS, participants engage in visual monitoring of environmental cues (block color/shape, container location) as well as the partner’s actions/social cues. These environmental and social cues will help in anticipating/predicting how to shape one’s own actions in response to the partner’s actions and environmental constraints (Semin and Cacioppo, 2009; Vesper et al., 2010). For example, the tester may begin to move their hand in the direction of a specific block and the child/adult monitoring the actions of the tester will pick up on these preparatory actions to accurately mimic the direction of tester’s actions. Additionally, participants will engage in moment-to-moment visual/reactive adaptations to account for changes in environmental cues and any corrective adjustments made by the tester as they continue to move in-synchrony (Semin and Cacioppo, 2009). As mentioned earlier, the STS region plays an important role in establishing visuomotor correspondence and would be activated as partners utilize joint attention or shared gaze to accurately monitor and match their own actions to that of their partner’s actions in a predictive or reactive manner (Molenberghs et al., 2010). It should be noted that the aforementioned regions do not work in isolation and are constantly interacting with each other and other cortical (e.g., primary, premotor and prefrontal cortices), and subcortical (e.g., cerebellum important for predictive control, etc.) structures (Gazzola and Keysers, 2009; Iacoboni, 2009; Caspers et al., 2010). Our current study findings fit with the above accounts in that both adult and child groups showed greater right STS and right IFG activation during the IPS conditions of the reach to cleanup task that also required greater spatial and temporal synchrony.

### Study Limitations

This preliminary study has some limitations in the study design. In terms of study design, we were unable to compare the brain activation patterns between the tester and the child. In the future, we plan to conduct a hyper scanning study to examine brain coherence between individuals engaging in IPS and imitation tasks. Additionally, there was some discrepancy in trials per condition completed by the adults (eight trials) vs. the children (six trials); however, we have calculated an average across trials for each condition and session. In terms of fNIRS data acquisition, we limited our analysis to 24 data channels over the lateral cortical surfaces and that did not capture prefrontal and motor cortex activity. Similarly, we were unable to implement the short-separation channels to account for skin-related hemodynamic responses as is implemented in other recent studies (Nguyen and Hong, 2016). Our subsequent studies have incorporated the full, 52-channel set up to collect lateral and prefrontal cortical activation. In terms of spatial registration, although we followed the international 10-20 system when placing probe sets, the variation of head size and probe placement could have added to the variability and inconsistency in the spatial registration of data channels. Finally, our study only reports the average hemodynamic responses; however, future studies should analyze detailed moment-to-moment changes in the hemodynamic response such as the initial dip in profile (see Hong and Naseer, 2016).

## Conclusions

In conclusion, the quality of IPS in school-age children did not reach adult levels, although their accuracy of reaching or attentional patterns appear similar to those of the adults. fNIRS was able to detect the developmental changes in cortical activation. Our results suggested that there is a pattern of greater right hemispheric activation when engaging in IPS tasks suggesting that IPS is a more complex behavior (above and beyond action observation or action execution) as it requires greater bilateral cortical activation. Moreover, children had less lateralization compared to adults during unilateral reach-cleanup motions suggesting a lack of transcallosal inhibition in children compared to adults. Lastly, the quality of adult synchrony correlated with activation in various cortical regions whereas the quality of child synchrony only correlated with activation in few cortical regions (i.e., right IFG and left IPL) providing further evidence for developmental differences in synchrony performance and its underlying control. In summary, there is a clear developmental trajectory for IPS behaviors as well as associated cortical activation patterns between childhood and adulthood. In the future, we plan to use these normative patterns to further understand atypical IPS behaviors and atypical cortical activation in children and adults with Autism Spectrum Disorder, a population that is known to have difficulties with imitation and IPS including difficulties in social and motor performance.

## Data Availability Statement

The datasets generated for this study are available on request to the corresponding author.

## Ethics Statement

The studies involving human participants were reviewed and approved by the University of Delaware’s Human Subjects Review Board. Written informed consent to participate in this study was provided by the participants’ legal guardian/next of kin.

## Author Contributions

W-CS contributed to this project through data collections, data analysis, and manuscript writing. MC, MH, and ST contributed to this project through recruitment, data collections, data analysis, and proofreading. KP was involved in the conception/planning phases of the project and assisted with proofreading. DT contributed to this project through the writing and implementation of his anchor-based spatial registration codes. He also assisted with the graphing of related data figures and writing of the spatial registration portion of the manuscript. AB provided oversight and help with recruitment, data collections, analyses, and manuscript writing.

## Conflict of Interest

The authors declare that the research was conducted in the absence of any commercial or financial relationships that could be construed as a potential conflict of interest.
