# Supplementary material for: Developmental Differences in Cortical Activation During Action Observation, Action Execution and Interpersonal Synchrony: An fNIRS Study
Source: Front Hum Neurosci. 2020 Mar 3;14:57. doi: 10.3389/fnhum.2020.00057 (PMC7062643; doi:10.3389/fnhum.2020.00057)
Supplement: Supplementary file 1 [file Data_Sheet_1.docx]

**Supplementary Materials.**

**Table S1 Assignment of channels to regions based on spatial registration data in TD children.**

For each channel, the spatial location in MNI’s coordinate system and the probability of covering different brain regions were shown. The channels were then assigned to a specific ROI. Channels 1 to 12 belonged to the left hemisphere and channels 13 to 24 belong to the right hemisphere. For channel-level analysis, we chose channels 1, 3 (Left/L) and 14, 17 (R/Right) for the SA regions, channels 2, 4, 5, 7 (L) and 13, 15, 16, 18 (R) for the PI regions, and channels 10, 11, 12 (L) and 20, 23, 24 (R) for the IA region. *In the case of adults (see Bhat et al., 2017), channels 6 and 8 on the left side and their right-sided homologues (channel 19 and 22) did not fall within the same ROIs, hence were excluded from analyses for both groups to keep channels consistent across groups. ** Channel 21 was not assigned to any region as the proportion of covering a certain region was not above 60%; hence, channel 21 and its homologue, channel 9 were excluded from the analysis for consistency.

Figure S1. Second to second blocked HbO_2_ data per condition and channel for *healthy adults*. Pink vertical line denotes the start of the stimulation period and the data shown to the right of the pink line are the 240 frames across stimulation (11–13 s) and post-stimulation baseline (13–11 s) periods.

Figure S2. Second to second blocked HbO_2_ data per condition and channel for *TD children*. Pink vertical line denotes the start of the stimulation period and the data shown to the right of the pink line are the 240 frames across stimulation (11–13 s) and post-stimulation baseline (13–11 s) periods.
